# Combination Therapy of Plasma Exchange and Rituximab to Treat Cicatricial Pemphigoid and Bullous Pemphigoid

**DOI:** 10.7759/cureus.19932

**Published:** 2021-11-27

**Authors:** Anuj Kunadia, Naveed Sami

**Affiliations:** 1 Dermatology, University of Central Florida College of Medicine, Orlando, USA

**Keywords:** autoimmune blistering diseases, rituximab, plasma exchange, bullous pemphigoid, cicatricial pemphigoid

## Abstract

The pemphigoid group of subepidermal autoimmune blistering diseases can affect both cutaneous and mucosal tissues. Therapy of this group of diseases, including cicatricial pemphigoid (CP) and bullous pemphigoid (BP), consists of systemic steroids and immunomodulatory agents. Recalcitrant cases have typically been treated with plasmapheresis or rituximab individually. This report describes two patients with severe, rapidly progressive CP and BP refractory to high-dosage systemic steroids and immunomodulatory agents. Both patients were treated with a combination of plasmapheresis and rituximab. In addition to these cases, one retrospective study showed the effectiveness of other immunosuppressants in combination with plasmapheresis in 17 patients with pemphigus refractory to corticosteroids and immunosuppressants alone. No major adverse events occurred in the study. Similar studies employing immunoadsorption and rituximab with various combinations of intravenous immune globulin (IVIg), corticosteroids, and other conventional immunosuppressants have shown promising results in other autoimmune blistering diseases. The successful response in the patients described here, as well as those described in the literature who underwent similar management, provides a possible combination treatment option for patients with severe, recalcitrant pemphigoid. A further trial with a larger group of pemphigoid patients is warranted.

## Introduction

The pemphigoid group of subepidermal autoimmune blistering diseases (ABDs) can affect both cutaneous and mucosal tissues and are further subclassified based on clinical features, histology, and immunopathology. Cicatricial pemphigoid (CP) primarily affects mucosal tissues and heals with scarring. Bullous pemphigoid (BP) more commonly presents with tense cutaneous blisters. Areas of involvement may overlap in both diseases. Conventional therapy of these diseases consists of systemic steroids and immunomodulatory agents. Patients who are unresponsive to conventional therapy have been treated with plasmapheresis or rituximab individually. This report describes two patients with severe BP and CP, respectively, each refractory to systemic steroids and immunomodulatory agents. Both patients were treated successfully with a combination of plasmapheresis and rituximab. 

## Case presentation

**Case 1**: A 50-year-old female (Patient 1) presented with large bullae diffusely present on the extremities and trunk along with oral, pharyngeal, nasal, and urogenital erosions. Physical examination did not reveal any mucosal scarring. The patient’s past medical history (PMH) was significant for diabetes mellitus, systemic lupus erythematosus, hepatitis C, chronic renal insufficiency, and cancer of the cervix and vagina in remission at the time of presentation. BP was confirmed with histopathological studies. The patient was unable to decrease the dosage of her prednisone below 80 mg/day without an exacerbation, despite concomitant treatment with mycophenolate mofetil, cyclosporine, azathioprine, dapsone, and intravenous immunoglobulin over 14 months. 

**Case 2**: A 42-year-old female (Patient 2) with no significant PMH presented with cutaneous blisters, dysphagia, and significant dyspnea. Physical examination revealed extensive erosions of her oral, pharyngeal, and urogenital mucosa. She also had significant conjunctival scarring, esophageal strictures, extensive nasal and laryngeal erosions, with scarring that resulted in dyspnea. Histopathological studies confirmed CP. She was unresponsive to prednisone (60 mg/day) and cyclophosphamide (100 mg/day) over six months.

**Management**: Both patients were treated with a combination of rituximab and plasmapheresis. The plasmapheresis protocol was administered according to the most recent Guidelines on the Use of Therapeutic Apheresis in Clinical Practice for pemphigus vulgaris [1]. The plasmapheresis protocol used five exchanges of one plasma volume with 5% albumin as replacement fluid every other day. Each patient received an equal dose of rituximab before the first (day one) and last (day 15) plasmapheresis session. Patient 1 received 500 mg based on the recommendation of her nephrologist. She was also cleared to receive rituximab by her hepatologist and oncologist due to the severity of her disease and failure of previous therapies. Patient 2 received 1 g of rituximab.

Clinical response in both patients was observed within two weeks of completing the treatment protocol. Patient 1 was able to successfully decrease the dosage of her prednisone to 20 mg/day over the next eight weeks with no other systemic steroid-sparing agent. Patient 2 was able to discontinue cyclophosphamide and reduce her prednisone dosage to 20 mg/day over 12 weeks. Her prednisone dose was further tapered down to 5 mg/day but due to ocular and laryngeal relapse after eight months, methotrexate (25 mg/week) was added to re-establish control.

## Discussion

Rituximab and plasmapheresis have each been individually used in both severe recalcitrant BP and CP with successful results [2-5]. Plasmapheresis and Protein A immunoadsorption (PAIA) have the potential for more rapid response as the autoantibodies are removed through filtration from the patient’s plasma. Conventional systemic oral immunosuppressive treatments have typically been used as adjuvant therapy to prevent both short- and long-term rebound of disease. 

Rituximab, a monoclonal antibody that targets B cells expressing CD-20, causes delayed depletion of these cells and has been successfully used in the treatment of ABDs. The delayed onset of Rituximab is compensated by its potential to induce long-term remission [6]. The two patients described, who had potentially fatal pemphigoid, responded well without notable adverse effects to combination therapy of plasmapheresis and rituximab. Prednisone was used to prevent a short-term rebound while rituximab was given as a steroid-sparing treatment, as well as long-term control of the disease. Rituximab was given after the patients failed to achieve control of their disease with conventional steroid-sparing oral treatments. The prednisone dosage was reduced over 8-12 weeks to allow the optimal therapeutic effects of rituximab which are generally seen after three months of initiating the infusions. Relapses can be observed with repletion of B cell counts six months after rituximab infusions. The second patient required methotrexate as a second steroid-sparing treatment after eight months of her initial rituximab treatment due to a relapse. Her insurance had denied an additional round of rituximab infusions. 

Severe and nonresponsive cases of other autoimmune bullous dermatoses such as pemphigus vulgaris and pemphigus foliaceus have been successfully treated with plasmapheresis, rituximab, or corticosteroids used independently [7]. There has been only one previous report of the treatment of bullous pemphigoid with the combination of plasmapheresis and rituximab. This case described a patient who had melanoma treated with nivolumab-induced bullous pemphigoid. The patient initially failed to respond to corticosteroid therapy and was subsequently treated with a combination of plasmapheresis and rituximab, resulting in near clearance of the patient’s lesions [8]. This patient was not treated with other conventional steroid-sparing treatments, presumably due to the rapidly progressing disease and history of melanoma. A literature review has also demonstrated the success of this approach in treating refractory cases of myasthenia gravis (MG), another autoimmune condition. Nine patients with refractory MG who were treated with plasmapheresis and rituximab showed an overall decrease in disease severity, long-term clinical improvement, and self-sufficiency [9]. 

While this case demonstrates the effectiveness of plasmapheresis and rituximab in two patients, one retrospective study showed the effectiveness of other immunosuppressants in combination with plasmapheresis in 17 patients with pemphigus refractory to corticosteroids and immunosuppressants alone. Combination therapy included plasmapheresis with cyclosporin A, mycophenolate mofetil, or methotrexate. Patients showed significantly decreased desmoglein antibodies and negative Nikolsky's sign with no new blisters appearing. Additionally, the dosage of corticosteroid was able to be tapered down rapidly over a range of 5-13 days. Of note, no major adverse events occurred to warrant termination of treatment [10]. Similar studies have shown promising results in conditions such as pemphigus vulgaris, pemphigus foliaceous, bullous pemphigoid, mucous membrane pemphigoid, and epidermolysis bullosa acquisita (Table 1). Treatments included immunoadsorption and rituximab with various combinations of intravenous immune globulin (IVIg), corticosteroids, and other conventional immunosuppressants [11-14]. Collectively, the described cases highlight the success of a therapeutic plan similar to that which was employed for the cases described in this report. Plasmapheresis and rituximab combination therapy effectively provides both a rapid and sustained improvement in patients’ autoimmune bullous diseases.

**Table 1:** Studies employing immunoadsorption and rituximab with various combinations of IVIg, corticosteroids, and/or other conventional immunosuppressants.

Case	Number of Patients	Treatment Protocol	Disease(s)	Initial Response	Adverse Effects	Clinical Outcome
Shimanovich et al. (2007) [11]	7	Combination of protein A immunoadsorption (PAIA), rituximab and conventional immunosuppressants; intravenous immune globulin (IVIg) if refractory	Pemphigus vulgaris (n=5); Pemphigus foliaceus (n=2)	Rapid decline in circulating autoantibodies with improvement of mucosal and cutaneous lesions within four weeks (n=7)	Not reported	Long term remission (n=3); Partial improvement (n=1); Resistant to initial therapy but good response to IVIg (n=3)
Kasperkiewicz et al. (2012) [12]	23	Immunoadsorption (IA) at initially three and later four-week intervals until lesions healed by 90%; 1000mg rituximab at weeks one and three; Intravenous dexamethasone pulses at first every three weeks then at increasing intervals in addition to daily azathioprine/mycophenolate mofetil	Severe Pemphigus	Rapid decline of circulating autoantibodies with improvement of pemphigus lesions in first weeks of therapy (n=23)	Severe, unspecified adverse events (n=2)	Complete remission (n=19); Minimal disease (n=1); Partial remission (n=3); Recurrence during long-term (mean 29 months) follow up (n=6)
Behzad et al. (2011) [13]	10	Initially immunoadsorption (IA) at four-week intervals; Rituximab either twice at 1000 mg or four times at 375mg; Corticosteroids were tapered according to the individual clinical status (over 12-month period)	Difficult to treat pemphigus vulgaris	Complete remission (n=8); Partial response (n=1); Unresponsive (n=1); Results at six months after first IA treatment	Not reported	Complete remission (n=6); Stable disease (n=1); Relapse (n=1); Remaining 2 patients not reported; Results at 12 months after first IA treatment
Kolesnik et al. (2014) [14]	15	Combination of protein A immunoadsorption (3-21 treatments) and rituximab (3-6 treatments); Additional low dose conventional immunosuppression	Pemphigus vulgaris (n=6); Bullous pemphigoid (n=3); Mucous membrane pemphigoid (n=3); Pemphigus foliaceus (n=2); Epidermolysis bullosa acquisita (n=1)	Rapid clinical improvement within first four weeks and decline of circulating autoantibody levels	Minimal long-term adverse effects	Complete/partial remission rates of 88%/12% in pemphigus diseases; Complete/partial remission rates of 71%/29% in subepidermal blistering diseases; Overall relapse rate was 13% (mean follow-up of 22 months)

## Conclusions

The success with the described patient cases, and those described in the literature who underwent similar therapy, provides a possible future combination treatment option for patients with severe rapidly progressive pemphigoid that is unresponsive to conventional oral treatment. Notably, a limitation of these findings includes the lack of a randomized control trial with a larger group of pemphigoid patients, which may be warranted to further study the efficacy of this treatment.
